# Cancer Diagnosis Using Terahertz-Graphene-Metasurface-Based Biosensor with Dual-Resonance Response

**DOI:** 10.3390/nano12213889

**Published:** 2022-11-03

**Authors:** Chunjian Tan, Shaogang Wang, Shizhen Li, Xu Liu, Jia Wei, Guoqi Zhang, Huaiyu Ye

**Affiliations:** 1 Electronic Components, Technology and Materials, Delft University of Technology, 2628 CD Delft, The Netherlands; 2 Engineering Research Center of Integrated Circuits for Next-Generation Communications, Ministry of Education, School of Microelectronics, Southern University of Science and Technology, Shenzhen 518055, China; 3 Bioland Laboratory, Guangzhou Regenerative Medicine and Health Guangdong Laboratory, Guangzhou 510005, China

**Keywords:** graphene metasurface, terahertz sensing, dual-resonance response, cancer diagnosis

## Abstract

Owing to the outstanding physical properties of graphene, its biosensing applications implemented by the terahertz metasurface are widely concerned and studied. Here, we present a novel design of the graphene metasurface, which consists of an individual graphene ring and an H-shaped graphene structure. The graphene metasurface exhibits a dual-resonance response, whose resonance frequency strongly varies with the geometrical parameters of the proposed metasurface, the carrier density of graphene, and the analyte composition. The transparency window, including width and position, can be artificially controlled by adjusting the geometrical parameters or the Fermi energy. Furthermore, the sensing parameters of the graphene metasurface for cancerous and normal cells are investigated, focusing on two factors, namely cell quantity and position on the metasurface. The simulated results clearly show that the theoretical sensitivity, figure of merit, and quantity of the graphene metasurface for breast cells reach 1.21 THz/RIU, 2.75 RIU${}^{-1}$, and 2.43, respectively. Our findings may open up new avenues for promising applications in the diagnosis of cancers.

## 1. Introduction

Cancer ranks as one of the leading causes of human morbidity and death globally [1,2]. It has also been a major impediment to increasing life expectancy in many countries [3]. The global cancer data for 2020 released by the World Health Organization (WHO) show that there were an estimated 19.29 million new cancer cases worldwide, including 9.96 million deaths [1,3,4]. In 2040, new cancer cases are projected to reach 28.4 million [3]. More specifically, the death tolls of the top five cancer types in 2020, i.e., lung, colorectum, liver, stomach, and breast cancer, were estimated as 1.796, 0.935, 0.83, 0.768 million, respectively [2,3]. The difficulty and types of cancer treatment depend upon the development level or staging of disease. Over the years, a wide variety of cancer detection techniques, such as radioimmunoassay [5], magnetic resonance imaging [6], and sonography [7], has been developed to satisfy numerous needs. The magnetic resonance and computed tomography methods are widely adopted for in vivo and in vitro cancer cell detection, but their limited resolutions are restricted by specific contrast agents [2,8,9]. Near-infrared fluorescence imaging is a method for detecting cancer cells or tissues because of its high tissue penetration and less background scattering [10]. However, the shortcomings of the obtained image and the near-infrared dyes, such as low spatial resolution, low detection sensitivity, and limited photostability, make it less promising for in vivo cancer imaging. In addition, the cancer cells/biomarker detection strategies, including time-resolved fluorescent assays [11], gas chromatography-coupled mass spectroscopy [12], electrochemiluminescent immunoassays [13], etc., are presently used for the initial screening of cancer patients in centralized and hospital-based laboratories. Although these techniques are improved regarding the specificity and sensitivity, there are still numerous hurdles, such as being time consuming, having high cost, requiring expensive and sophisticated equipment, requiring a cumbersome pretreatment and analysis process, and the inability to detect cancer at early stages [14,15]. Therefore, to seek safer and more efficient cancer detection and treatment methods is of the utmost importance for cancer prevention and disease management [16], especially in the diagnosis of cancers.

Terahertz (THz) spectroscopy, electromagnetic waves within the frequency range 0.1–10 THz, has attracted increasing attention from scientists for its promising applications in biomedical analytics, detecting, imaging, trapping, and sensing due to its advantages of being label-free, non-contact, and non-destructive [17,18,19,20,21]. In the THz domain, a large amount of complex biological components, including biological molecules, cells, and tissues, have collective vibrational and rotational modes that correspond to a unique characteristic spectrum [22,23,24]. Owing to the lack of proper materials with a strong response to the THz regime, research and application in the THz region have been hindered to a great extent [25]. In order to overcome this technical barrier, numerous efforts on metamaterials and metasurfaces have obtained abundant achievement [26,27,28,29,30,31]. The metamaterials possess strong electromagnetic field confinement and sharp resonance features [32,33,34,35], which can strengthen the THz waves–matter interaction and heighten the sensing performance in bio-detection application. Usually, the metamaterials are composed of metallic structures with a size scale smaller than the incident wavelength. Unfortunately, the intrinsic drawback (i.e., Ohmic losses) of these conventional metallic metamaterials inevitably degrades their performance of the optical responses [36,37], such as the quality factors of resonance and the efficiency [38]. Thus, new metamaterials are being sought for THz biosensing. Inspired by the nanomaterials and nanotechnologies, carbon-based materials have drawn much research interest as metamaterials of THz devices because they possess a rich variety of physical properties compared to conventional metamaterials, such as ultra-broadband optical absorption and response [39,40,41].

Graphene, as the most promising carbon-based nanomaterial for realistic application, has been applied favorably as optical imaging or sensing elements because of its high specific surface area [42], tunable optoelectronic property [43], and excellent biocompatibility [44]. In particular, the optical conductivity of graphene presents a Drude-like conductance behavior in the THz range, being changed with controlled free-carrier densities [45,46]. It is suitable for dynamical tenability. In order to gain the outstanding optoelectronic features, various flat structure designs in graphene have been proposed [47,48,49]. Nevertheless, it has been found that only one resonant optical mode can be induced by those graphene structures, which restricts the light–matter interaction in THz biosensing [50]. Therefore, it is of interest to apply multiple resonance scheme to enhance the interaction between light and matter. Several proposed multiresonance graphene structures have successfully confirmed that the multiresonance scheme can improve the sensing performance and strengthen the light–matter interaction [25,51,52]. Importantly, it makes multiband THz biosensing possible. The tunability of multiple channels and multiplexing properties in graphene plays a critical role in systems, including spectroscopy, sensors, and absorbers [53]. Graphene-based metamaterials or metasurfaces are usually polarization sensitive and invalid for non-normal incidence, leading to the degradation and disappearance of the sensing capability [25].

In this work, we propose a planar graphene metasurface, which is composed of a circular graphene ring and an H-shaped graphene structure. A feature of dual-resonance response is found in the transmission spectra, being expected for biosensors with high sensing performance. Such a feature can be controlled by adjusting the spacing size. The transparency window is dependent on the structural parameters of the metasurface and the carrier density of graphene. Moreover, the sensing performance of our proposed graphene metasurface for breast cells is also evaluated in our work. The results present that the parameters of breast cells, including cell types, number of cells, and position of cells on the graphene metasurface, have an obvious influence on the transmission spectra and sensing parameters of the proposed graphene metasurface.

## 2. Theoretical Model and Methodology

**Figure 1:**
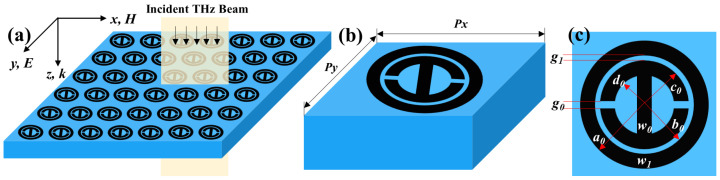
Schematic diagram of the proposed graphene-based metasurface on SiO${}_{2}$ substrate. (**a**) Periodic structure where the incident THz waves with y-polarization is along the *z*-axis. (**b**) A unit cell and (**c**) its top view with geometrical parameters. $a_{0}$ = 18 μm, $b_{0}$ = 12 μm, $c_{0}$ = 11 μm, $d_{0}$ = 7 μm, $g_{0}$ = 1 μm, $g_{1}$ = 1 μm, $w_{0}$ = 4 μm, and $w_{1}$ = 6 μm. The periodicity is set to 50 μm in both *x* and *y* directions.

The schematic structure of the graphene metasurface is illustrated in Figure 1. The unit cell comprises a toroidal ring and an H-shaped pattern of graphene monolayer positioned on the surface of a 500-μm-thick SiO${}_{2}$ substrate with the refractive index *n* = 1.956 [54]. The graphene-based unit cells are arranged in a periodic array in the *x*-*y* plane, as depicted in Figure 1a. *P* represents the periodicity of the array patterns. The spacing distance of the concentric inner and outer graphene rings and the corresponding ring width of the inner and outer rings are designated as $g_{1}$, $w_{0}$ and $w_{1}$, respectively. The gap size of the H-shaped pattern is indicated as $g_{0}$. In addition, the width of the graphene strip positioned at the center of the inner graphene ring is the same as that of the two graphene rings. The geometrical parameters of the unit cell are described specifically in Figure 1b. Typically, graphene films are synthesized by using chemical vapor deposition techniques and can be patterned lithographically to fabricate the designated graphene geometries at controlled locations [55]. One study showed that the laser-induced graphene method can be used in the fabrication of graphene metasurfaces and metamaterials [56].

In our work, the thickness of monolayer graphene is set as 0.35 nm [57]. The incident plane wave with *y*-polarization propagates vertically in a direction paralleling to the *z*-axis to the graphene metasurface. The transmission spectrum as a function of the incident wavelengths and the electric field distributions at resonance peaks are calculated in the full-wave electromagnetic simulator, COMSOL Multiphysics. In order to obtain the response characteristic of the graphene metasurface in the THz domain, we utilize an effective surface conductivity approach to characterize the graphene monolayer. Theoretically, the surface conductivity of the graphene can be predicted within the Kubo approximation, being composed of the intra-band and inter-band contribution of the electron transitions. According to the Kubo formula, the corresponding surface conductivity and electron transition contributions are as follows [58]:

(1) $$\sigma \left(\omega ,\Gamma ,\mu_{c},T\right)=\sigma_{intra}\left(\omega ,\Gamma ,\mu_{c},T\right)+\sigma_{inter}\left(\omega ,\Gamma ,\mu_{c},T\right)$$

where $\sigma_{intra}$ and $\sigma_{inter}$ are expressed as:

(2) $$\sigma_{intra}\left(\omega ,\Gamma ,\mu_{c},T\right)=\frac{ie^{2}k_{B}T}{\pi \hbar^{2}\left(2\pi v\lambda^{-1}+i\tau^{-1}\right)}\left[\frac{\mu_{c}}{k_{B}T}+2ln\left(exp\left(-\frac{\mu_{c}}{k_{B}T}\right)+1\right)\right]$$

(3) $$\sigma_{inter}\left(\omega ,\Gamma ,\mu_{c},T\right)=\frac{ie^{2}}{4\pi \hbar }ln\left[\frac{2|\mu_{c}|-\left(2\pi v\lambda^{-1}+i\tau^{-1}\right)\hbar }{2|\mu_{c}|+\left(2\pi v\lambda^{-1}+i\tau^{-1}\right)\hbar }\right]$$

in which *v* = 299,792,458 m/s is the velocity of light in vacuum, λ is the incident wavelength, $k_{B}$ = 1.3806488 × $10^{-23}$, J/K is the Boltzmann’s constant, T = 300 K, *ℏ* = h/2π is the reduced Plank constant, *e* = 1.602176565 $\times \;10^{-19}$, and C is the electron charge. The chemical potential is determined by $\mu_{c}=\hbar v_{F}\sqrt{\pi n_{s}}$ in which $n_{s}$ is the carrier concentration [59]. Unless otherwise stated, $n_{s}$ = 7 × $10^{13}$ cm${}^{-2}$ is used for all calculations, which is achievable under realistic conditions [60]. Moreover, the electron–phonon relaxation time is calculated by $\tau =\left(\mu \mu_{c}\right)/\left(ev_{F}^{2}\right)$, where $\mu =10^{4}$ cm${}^{2}$·$V^{-1}$·$s^{-1}$ is the carrier mobility and $v_{F}=10^{6}$ m ·$s^{-1}$ is the Fermi velocity. In the THz and mid-infrared regime, the surface conductivity of graphene is dominated by the intra-band transition due to $k_{B}$T ≪ $|\mu_{c}|$. Therefore, the inter-band transition is neglected, and the surface conductivity of graphene can be simplified as [58]

(4) $$\sigma =\frac{ie^{2}\mu_{c}}{\pi \hbar^{2}\left(2\pi v\lambda^{-1}+i\tau^{-1}\right)}$$

## 3. Results and Discussion

**Figure 2:**
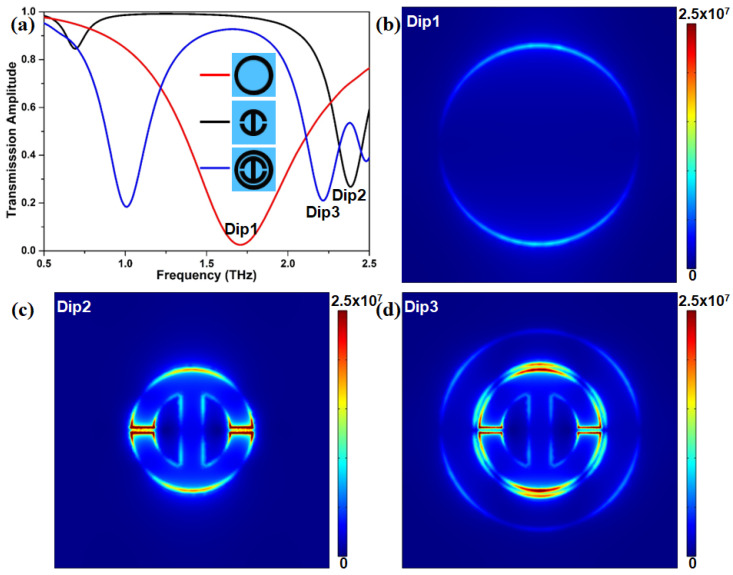
(**a**) Normalized THz transmission amplitudes of the graphene ring, H-shaped structure, and both of them. (**b**,**d**) Distributions of electric field at frequency points Dip1, Dip2, and Dip3. These three points are corresponded to the frequencies: (**b**) 1.70 THz, (**c**) 2.38 THz, and 2.2 THz, respectively.

Before beginning to systematically analyze the sensing performance of graphene-based helipad-shaped devices, the response mechanism of the proposed graphene metasurface in the desired THz region should be discussed. The amplitude transmission spectra of the circular graphene ring, the H-shaped pattern, and the combined structure are presented in Figure 2a. It clearly illustrates that there is only a resonance transmission dip located at 1.70 THz within the investigated THz window when only the outer graphene ring exists. Meanwhile, for the case of only the H-shaped structure, there are two transmission dips located at 0.69 THz and 2.38 THz, respectively. The latter is more remarkable than the former. As a result, these two graphene microstructures can directly couple with the incident light in the THz region, acting as the bright mode. When these two graphene microstructures are integrated together, a distinct transmission curve with two resonance dips is obtained. These two dips are located at 1.00 THz and 2.20 THz, respectively. This result reveals that our proposed graphene metasurface can efficiently excite double resonance responses. To deeply decipher the underlying physical mechanism of the dual resonance phenomenon, the electric field distribution at each resonant dip labeled “Dip1”, “Dip2”, and “Dip3” in Figure 2a is studied as displayed in Figure 2b–d. We find that the electric field in Figure 2b mainly distributes at the outer edge of the graphene ring with an extremely weak intensity. For the H-shaped microstructure in Figure 2c, the electric field distributes at the edge of the pattern, and the intensity at the two gaps is much higher than that at the other portions. Furthermore, for the combined microstructure, as presented in Figure 2d, the electric field at the inner edge of the circular graphene ring and the outer edge of the H-shaped microstructure is enhanced. Interestingly, the electric field at the two gaps of the H-shaped microstructure becomes weaker. This convincingly shows that there is an energy transference of the electric field in the proposed graphene metasurface, originating from the near field coupling between the bright and dark modes [61]. Ultimately, a destructive interference occurs, which leads to a different transparency window.

**Figure 3:**
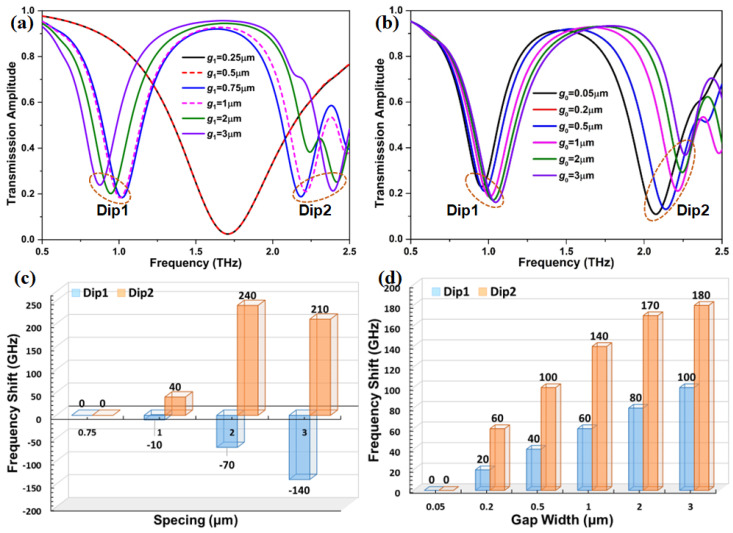
Transmission spectrum of the graphene metasurface as a function of (**a**) the spacing between the circular graphene ring and the H-shaped microstructure, and (**b**) the gap width of the H-shaped microstructure. (**c**,**d**) The frequency shift versus different spacing and gap widths in the proposed metasurface.

In order to explore the tunable window of the proposed graphene metasurface, we investigate the influence on it through adjusting the geometric parameters as depicted in Figure 3. $g_{0}$ denotes the gap width of the H-shaped microstructure, and $g_{1}$ is the spacing between the inner edge of the circular graphene ring and the outer edge of the H-shaped microstructure along the radial direction. It can be found that when the spacing $g_{1}$ is 0.25 μm and 0.5 μm, these two distinct microstructures produce almost the same transmission spectra, and their dips with a transmittance of 2.5% appear at 1.7 THz. A dual resonance phenomenon can be clearly observed in the same THz region when $g_{1}$ is increased to 0.75 μm, as shown in Figure 3a. Two transmission dips in the transmission spectra are located at 1.02 THz and 2.18 THz, respectively, and a noticeable transparency window appears between these two dips. It can be also observed that when $g_{1}$ further increases from 0.75 μm to 3 μm, the feature of the dual resonance response still persists, and the transparency window becomes wider. Meanwhile, the spectral position of the transmission Dip1 exhibits a red-shifted behavior, while a blueshift is found for the transmission Dip2. The displacement of transmission Dip2 is significantly larger than that of transmission Dip1 when $g_{1}$ is 2 μm and 3 μm. This result is further confirmed by the frequency shift in Figure 3c. The transmission spectra under different $g_{0}$ is illustrated in Figure 3b. With the increasing of $g_{0}$ from 0.05 μm to 3 μm, each transmission dip is blueshifted, and the dual resonance feature is not unaffected. However, there is an obvious difference in the displacement of these two dips. As seen in Figure 3d, compared with the transmission Dip1, the displacement of the transmission Dip2 is more than two times its displacement at a given $g_{0}$. Moreover, the transparency window first becomes larger and then remains unchanged when $g_{0}$ is increased to 3 μm from 0.05 μm. For the line width of two rings, the transmission spectra directly show that the line width of two rings has a significant influence on the transmission spectea of the graphene metasurface, as displayed in Figure S1. According to the results, we conclude that the dual resonance feature and the transparency window can be individually controlled by modulating the structural parameters of the graphene metasurface.

**Figure 4:**
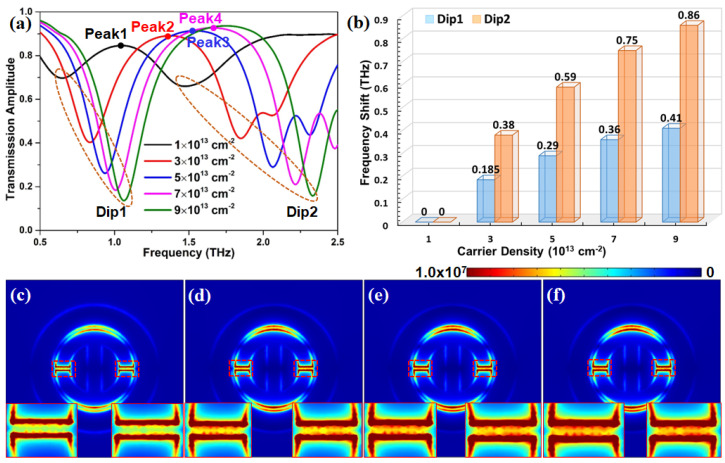
(**a**) Transmission spectrum with different carrier densities. (**b**) The frequency shifts versus various carrier densities at two major resonance dips of the proposed graphene metasurface. (**c**–**f**) Distributions of the electric field for the corresponding transmission peaks as denoted in panel (**a**).

One of the most interesting features of graphene is that its conductivity depends largely on the chemical potential, which can be controlled by the carrier density [62]. Additionally, in addition to the geometric parameters, the carrier density is another critical parameter that affects the performance of the proposed metasurface. Figure 4a shows the effect of carrier density (chemical potential) on the transmission spectra of our proposed graphene metasurface. It is clearly found that the spectral position of all transmission dips moves into a higher frequency regime with the increasing of the carrier density from 1 × $10^{13}$ cm${}^{-2}$ to 9 × $10^{13}$ cm${}^{-2}$. This means that an increasing carrier density induces an obvious blueshift phenomenon. In Figure 4b, when the carrier density of the graphene metasurface increases from 1 $\times \;10^{13}$ cm${}^{-2}$ to 9 $\times \;10^{13}$ cm${}^{-2}$, the frequency shifts of Dip1 and Dip2 present a gradually increasing trend. Meanwhile, the frequency shift of transmission Dip2 is almost two times that of transmission Dip1. That result indicates that the variation of the doping can control the position and dimension of the transparency window. Figure 4c–f depict the electric field distribution at the transmission peak with different carrier densities. We can find that the electric field mainly distributes the two gaps of the H-shaped microstructure and the top-/bottom-spacing defined as the distance between the inner edge of the graphene ring and the outer edge of the H-shaped microstructure, ignoring the carrier density effect. When the carrier density increases from 1 × $10^{13}$ cm${}^{-2}$ to 7 × $10^{13}$ cm${}^{-2}$, the electric field on the same positions becomes stronger. However, it should be noted that there is a minute deference in the intensity of the electric field as the carrier density of graphene is increased to 7 × $10^{13}$ cm${}^{-2}$ from 5 × $10^{13}$ cm${}^{-2}$, as displayed in the insert plots of Figure 4e,f, which is further confirmed by 3D electric field distribution in Figure S2. That means that with further increasing carrier density of graphene, the electric field intensity of the proposed metasurface may arrive at a threshold. Therefore, it is extremely important to select a proper carrier density for designing graphene metasurface with excellent properties.

**Figure 5:**
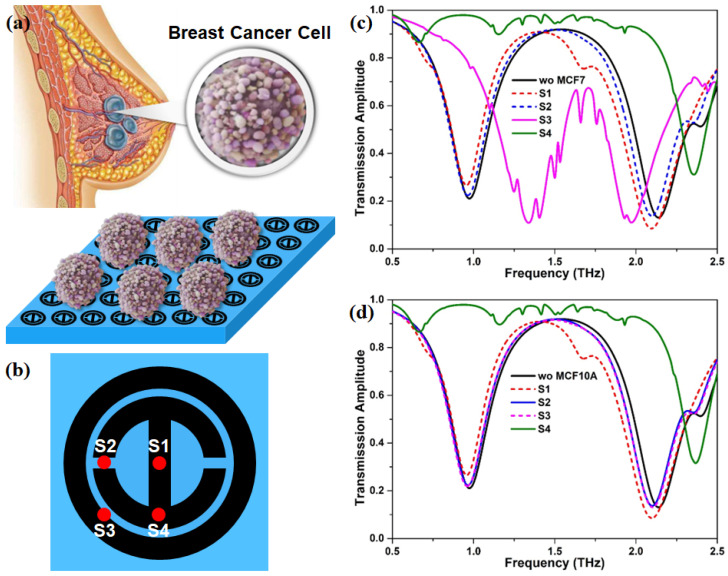
(**a**) The schematic diagram of the breast cancer cell and their sensing process. (**b**) The possible sites of MCF10A and MCF7 on the graphene metasurface. (**c**,**d**) The transmission spectra of single MCF7 and MCF10A on different sites in panel (**b**).

In order to investigate the sensing performance of the proposed graphene metasurface, two breast cells, namely, breast normal cell (MCF10A) and breast cancer cell (MCF7), are chosen as the target analytes. In actual experiments, analytes are not evenly covered on the sensing region. Thus, in this simulation, MCF10A and MCF7 breast cells are supposed to be in discrete hemispheres, where their diameters are set to 18.7 μm and 18.9 μm, respectively [63], and the corresponding refractive indexes are 1.387 and 1.401 [64], respectively. As illustrated in Figure 5b, four possible sites of MCF10A and MCF7 on the proposed graphene metasurface are considered to uncover the underlying effect of different sites on the transmission response. In the case of single MCF7, we find that the transmission spectrum presents a significant redshift when MCF7 is located at the S1 and S2, but the amount of the redshift at the S1 is larger than that at the S2. Interestingly, the transparency window moves toward a higher frequency region as MCF7 appears at the S3, and it becomes narrower. Meanwhile, the transmission peak has a large decrease, as displayed in Figure 5c. It is unexpected that the first dip near 0.75 THz almost disappears when MCF7 is arranged at the S4, and the second dip in the vicinity of 2.5 THz has an obvious blueshift. By inspecting Figure 5d, we also observe that the response trend of the transmission spectrum is similar to that of the MCF7 case when MCF10A is at S1, S2, and S4. Nevertheless, the transmission spectrum with MCF10A at S3 is different from that of MCF7 with the same site, but almost the same as that with MCF10A at S2. Therefore, the breast cancer cells can be effectively identified by their transmission spectrum.

**Figure 6:**
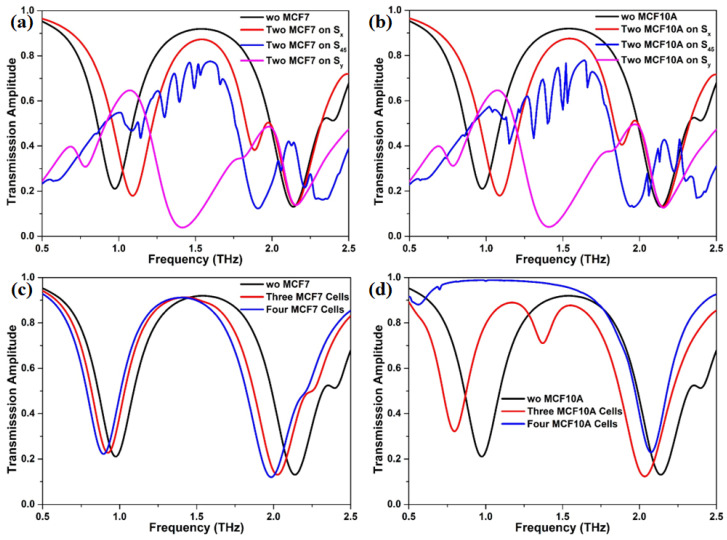
The corresponding transmission spectra of the proposed metasurface with (**a**) two MCF7, (**b**) two MCF10A, (**c**) three and four MCF7, and (**d**) three and four MCF10A.

Considering the actual situation, we also investigate the effect of number of breast cell on the transmission response, as shown in Figure 6. It should be pointed out that three possible scenarios about the distribution of two MCF7 or two MCF10A on the graphene metasurface, namely lied along the *x* axis, oblique direction (45${}^{\circ }$ above the horizontal), and *y* axis, are taken into account. The transmission spectrum in Figure 6a,b clearly presents that the distribution position of two MCF7 or two MCF10A has an obvious effect on the response of incident THz beam. Each scenario produces its own distinct transmission spectrum. Meanwhile, we observe that there is little difference in the transmission spectrum between the two-MCF7 case and two-MCF10A case, which could result in the inability to effectively recognize breast cancer cells from breast normal cells. However, the transmission spectra exhibit different responses when the number of breast cell is changed to two from one. In contrast to the two-cells case, the response shape of the transmission spectra of the three-/four-MCF7 case is nearly the same as that without breast cells, as plotted in Figure 6c. The whole transmission spectrum moves toward the lower frequency region with the increasing of the number of MCF7 from three to four. Curiously, when the number of MCF10A is increased to three from two, a new dip that lies in the range of 1.25 THz and 1.50 THz is excited, as illustrated in Figure 6d. The amount of redshift of the dip near 0.75 THz is much bigger than that of the dip near 2.0 THz, which means that the dual-resonance response may be favorable for detecting cancer cells with a low density. For the four-MCF10A case, the first dip of the transmission near 0.5 THz practically disappears, but its second dip still is preserved. An expected redshift is observed in the transmission spectra. Overall, the difference in THz response between MCF7 and MCF10A under the same conditions is in favor of diagnosing cancer cells.

As the important elements of the biosensors, sensitivity (S), figure of merit (FOM), and quality-factor (Q) can effectively characterize the sensing quality. These elements are defined as [65]

(5) $$S=\frac{△f}{△n}\left(\mathrm{THz}/\mathrm{RIU}\right)$$

(6) $$FOM=\frac{S}{Full-WidthatHalf-Maximum}$$

(7) $$Q=\frac{f_{res}}{Full-WidthatHalf-Maximum}$$

where △f is the difference between the reference frequency without breast cell and the resonance frequency with MCF7 or MCF10A. △n is the variation of the refractive index relatives to the air (*n* = 1). $f_{res}$ is the resonance frequency without or with the breast cells. Notably, these sensing parameters are calculated at $g_{0}$ = 0.2 μm and $d_{0}$ = 1.0 μm. Since the transmission peak almost disappears, the sensing parameters of the two-MCF7 case at S4, the two-MCF10A case at S4, and four-MCF10A case are not considered in this discussion. The $f_{res}$, S, FWHM, FOM, and Q of our proposed graphene metasurface without and with breast cell are listed in Table 1. Among all investigated cases, the S, FOM, and Q of the proposed graphene metasurface with two MCF10A at at S${}_{y}$ are highest, namely, 1.21 THz/RIU, 2.75 RIU${}^{-1}$, and 2.43, respectively. Similarly, it can also be found that when two-MCF7 is arranged at S${}_{y}$, the graphene metasurface exhibits relatively high sensing parameters, namely, 1.17 THz/RIU, 2.66 RIU${}^{-1}$, and 2.43, which are smaller than that of the two-MCF10A case at the same site. Furthermore, when one MCF7 appears at S3, the graphene metasurface possesses a FOM of 1.06 RIU${}^{-1}$ and a Q of 4.26, but its S factor only is 0.42 THz/RIU. In the meantime, this behavior also occurs in the three-MCF10A case, and the corresponding parameters are 0.96 THz/RIU, 1.53 RIU${}^{-1}$, and 1.87, respectively. These findings reveal that the site of the breast cell on the graphene metasurface has a noticeable influence on the sensing performance.

Ultimately, to better evaluate the sensing performance of our proposed graphene metasurface for normal and cancerous breast cells, its sensing parameters are compared to that reported in previous studies, as listed in Table 2. In comparison, the theoretical sensitivity of the proposed graphene metasurface is relatively higher than that reported in some literatures. However, it still needs to be improved in terms of structural design or materials compared to sensors with ultra-high sensitivity. Similarly, its FOM and Q values are moderate. These two parameters are more focused on in the optimization design due to the relatively lower values. Although there are some improvements to be performed in the proposed graphene metasurface, its sensitivity is sufficient to identify the normal and cancerous tissue cells.

## 4. Conclusions

In summary, we numerically and theoretically investigate the graphene metasurface, which consists of an individual graphene ring and a H-shaped graphene microstructure. It is found that this metasurface presents a behavior of dual-resonance response, which may be favorable for improving the sensing capacity of human cancers. The dual-resonance feature is controlled by adjusting the spacing size, but is unchanged by the gap size of the H-shaped microstructure and the carrier density of graphene. Moreover, we notice that the size and position of the transparency window are highly sensitive to the geometrical parameters of the proposed metasurface and the carrier density of graphene. Thus, the transparency window can be adjusted over a broad frequency range. The sensing performance is also studied in depth, and it is clearly shown that the sensing parameters are dependent on the cell type, cell count, and the position of cells on the graphene metasurface. Such a graphene metasurface achieves an acceptable S, FOM, and Q, and the corresponding values are 1.21 THz/RIU, 2.75 RIU${}^{-1}$, and 2.43, respectively. Our work may offer new possibilities for exploiting graphene or other 2D materials as sensing materials for cancer diagnosis.

## Figures and Tables

**Table 1:** Characteristics of the proposed graphene metasurface without and with breast cells.

Analyte	*n*	Num.	Site	$f_{\mathrm{res}}$ (THz)	△f (THz)	S	FWHM (THz)	FOM (RIU${}^{-1}$)	Q
air	1.0			1.54			0.92		1.67
MCF7	1.401	one	S1	1.40	0.14	0.35	0.89	0.39	1.57
			S2	1.50	0.04	0.10	0.90	0.11	1.67
			S3	1.71	0.17	0.42	0.40	1.06	4.26
		two	S${}_{x}$	1.54	0.00	0.00	0.65	0.00	2.37
			S${}_{45}$	1.52	0.02	0.05	1.02	0.05	1.49
			S${}_{y}$	1.07	0.47	1.17	0.44	2.66	2.43
		three		1.42	0.12	0.30	0.87	0.35	1.64
		four		1.40	0.14	0.35	0.85	0.41	1.66
MCF10A	1.387	one	S1	1.41	0.13	0.34	0.89	0.38	1.58
			S2	1.51	0.03	0.08	0.90	0.09	1.68
			S3	1.49	0.05	0.13	0.90	0.14	1.65
		two	S${}_{x}$	1.54	0.00	0.00	0.66	0.00	2.33
			S${}_{45}$	1.52	0.02	0.05	1.04	0.05	1.46
			S${}_{y}$	1.07	0.47	1.21	0.44	2.75	2.43
		three		1.17	0.37	0.96	0.62	1.53	1.87

**Table 2:** Parametric comparison of the reported THz metamaterials or metasurfaces.

Microstructure	S (THz/RIU)	FOM (RIU${}^{-1}$)	Q	Ref.
Present work	1.21	2.75	2.43	
MEMS-based metamaterial	0.379	63.83	66.01	[66]
All-polymeric THz sensor	3.34	8857	23,670	[67]
GMA based sensor	2.372	64.62	179.95	[68]
Split-ring resonator with four-gaps	0.285	1.88	6.6	[69]
Electric split-ring metamaterial	0.457	35.47	40.65	[70]
Black phosphorus-based nanostructure	1.06	0.166	∼	[71]
3D graphene metastructure	0.96	∼	∼	[72]

## Data Availability

The data is available on reasonable request from the corresponding author.

## Supplementary Materials

The following supporting information can be downloaded at: https://www.mdpi.com/article/10.3390/nano12213889/s1, Figure S1: (a) Simulated transmission spectra and (b) resonance frequency of the graphene metasurface for different line widths of two graphene rings. Note that black, red, blue, olive, violet, and brown colors are denoted as $w_{0}$ = 4 μm, $w_{1}$ = 6 μm; $w_{0}$ = 4 μm, $w_{1}$ = 4 μm; $w_{0}$ = 4 μm, $w_{1}$ = 2 μm; $w_{0}$ = 2 μm, $w_{1}$ = 2 μm; $w_{0}$ = 2 μm, $w_{1}$ = 4 μm; $w_{0}$ = 2 μm, $w_{1}$ = 6 μm, respectively; Figure S2: The 3D electric field of the graphene metasurface with the carrier density of (a) 1E13 cm${}^{-1}$, (b) 3E13 cm${}^{-1}$, (c) 5E13 cm${}^{-1}$, and (d) 7E13 cm${}^{-1}$.
