# Supplementary material for: Cancer Diagnosis Using Terahertz-Graphene-Metasurface-Based Biosensor with Dual-Resonance Response
Source: Nanomaterials (Basel). 2022 Nov 3;12(21):3889. doi: 10.3390/nano12213889 (PMC9654090; doi:10.3390/nano12213889)
Supplement: Supplementary file 1 [file nanomaterials-12-03889-s001.zip › nanomaterials-2003479-supplementary.pdf]

Supporting information

Cancer diagnosis using terahertz graphene metasurface based biosensor with dual-resonance response

Chunjian Tan[1,2,‡], Shaogang Wang[1,2,‡], Shizhen Li [2], Xu Liu[a,b], Jia Wei[3], Guoqi Zhang[1,*], Huaiyu Ye[2,*]

[1.]Electronic Components, Technology and Materials, Delft University of Technology, 2628 CD Delft, The Netherlands.

[2.]Engineering Research Center of Integrated Circuits for Next-Generation Communications, Ministry of Education, School of Microelectronics, Southern University of Science and Technology, Shenzhen 518055, P. R. China.

[3.] Bioland Laboratory, Guangzhou Regenerative Medicine and Health Guangdong Laboratory, Guangzhou 510005, P. R. China.

* Correspondence: G.Q.Zhang@tudelft.nl and yehy@sustech.edu.cn

‡ These authors contributed equally to this work.

In Figure S1(a), we find that there is a red-shift with reduction of transmisson strength for Dip1 and Dip2 when w_0=4 μm and simultaneously w_1 changes from 6 μm to 2 μm. In ccontrast, there is a blue-shift behavior for Dip1 and Dip2 when w_0 is fixed as 2 μm and w_1 increases from 2 μm to 6 μm except for Dip2 at w_0=2 μm and w_1=6 μm. Besides, the transmission window between Dip1 and Dip2 for w_0=2 μm is larger than that of w_0=4 μm. Therefore, the line width of two rings has a significant influence on the transmission spectea of the graphene metasurface.

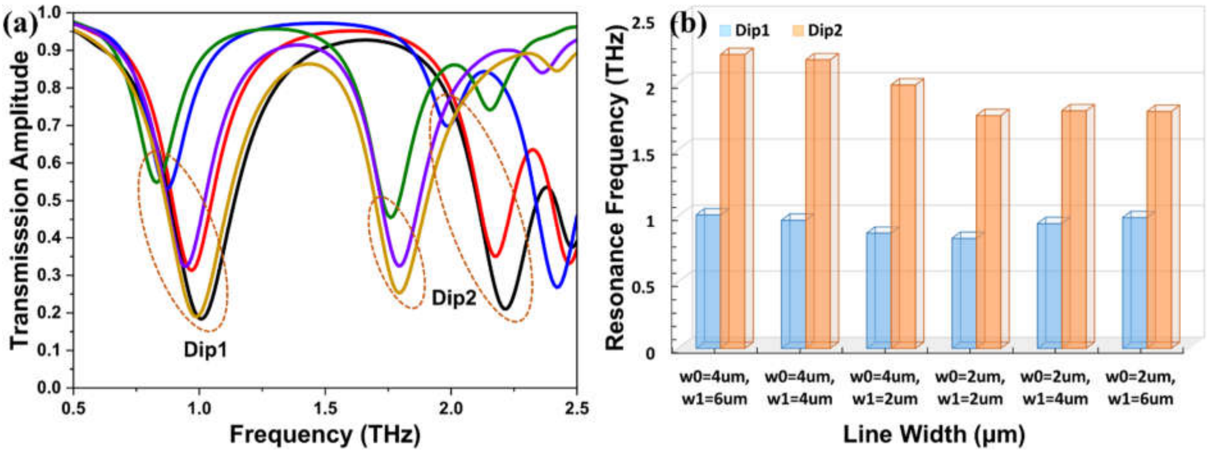

Figure S1. (a) Simulated transmission spectra and (b) resonance frequency of the graphene metasurface for different line widths of two graphene rings. Note that black, red, blue, olive, violet, and brown colors are denoted as w_0=4 μm, w_1=6 μm; w_0=4 μm, w_1=4 μm; w_0=4 μm, w_1=2 μm; w_0=2 μm, w_1=2 μm; w_0=2 μm, w_1=4 μm; w_0=2 μm, w_1=6 μm, respectively.

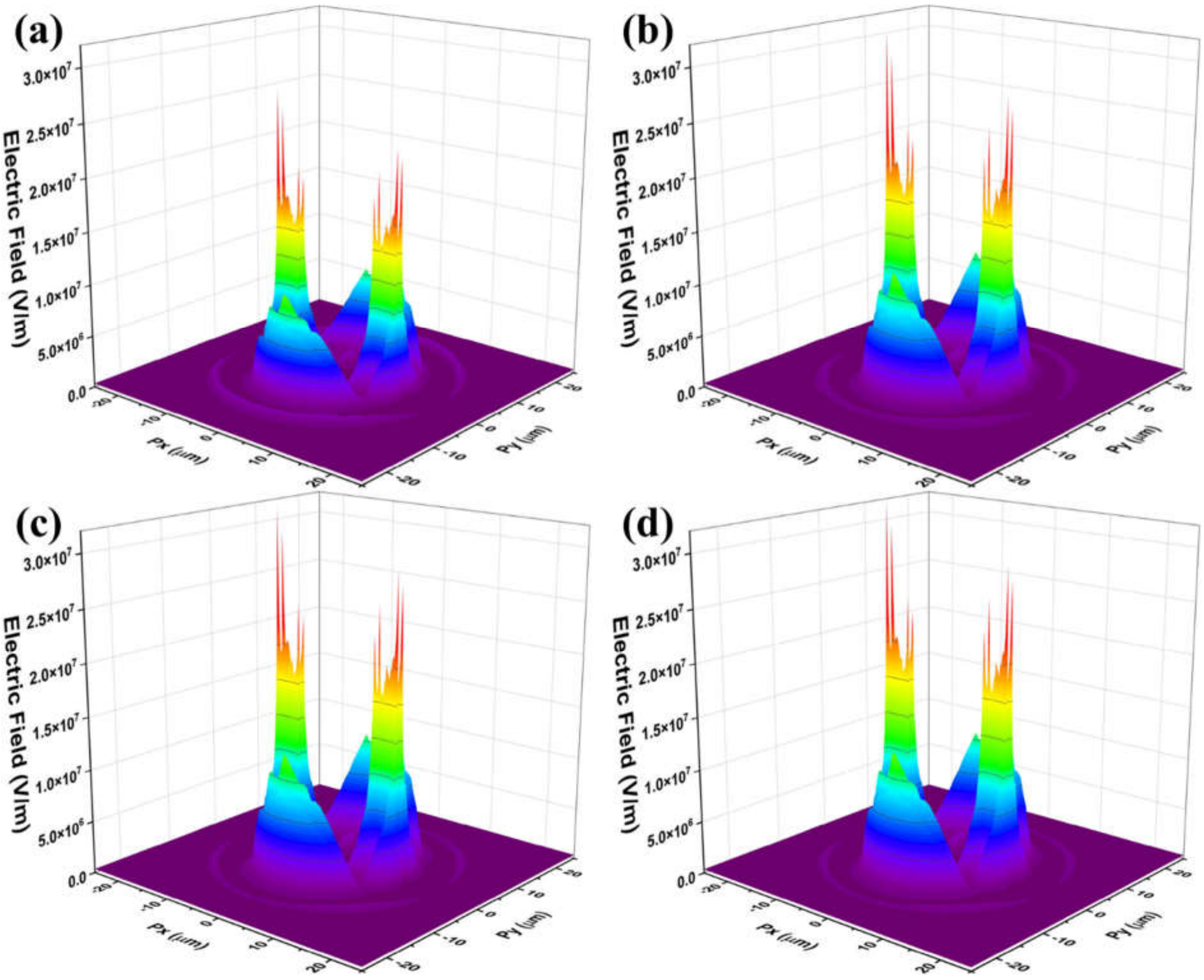

Figure S2. The 3D electric field of the graphene metasurface with the carrier density of (a) 1E13 cm^{-1}, (b) 3E13 cm^{-1}, (c) 5E13 cm^{-1}, and (d) 7E13 cm^{-1}.
